# HCV infection characteristics, treatment uptake and outcomes in patient with diabetes mellitus

**DOI:** 10.1186/s12902-022-01198-x

**Published:** 2022-11-12

**Authors:** Marina Angel, Yelena Petrosyan, Mary-Anne Doyle, Curtis Cooper

**Affiliations:** 1 Ottawa Hospital Research Institute, G12-501 Smyth Road, Ottawa, ON K1H 8L6 Canada; 2 Department of Medicine, University of Ottawa, Ottawa, ON K1H 8L6 Canada; 3 School of Epidemiology and Public Health, University of Ottawa, Ottawa, ON K1G 5Z3 Canada

**Keywords:** Hepatitis C, Diabetes, Direct Acting Antiviral Treatment

## Abstract

**Background:**

The interplay between HCV, DM, and DAA therapy is poorly understood. We compared HCV infection characteristics, treatment uptake, and treatment outcomes in patients with and without DM.

**Methods:**

A retrospective cohort study was conducted using data from The Ottawa Hospital Viral Hepatitis Program. Statistical comparisons between diabetes and non-diabetes were made using χ^2^ and t-tests. Logistic regression analyses were performed to assess predictors of DM and SVR.

**Results:**

One thousand five hundred eighty-eight HCV patients were included in this analysis; 9.6% had DM. Patients with DM were older and more likely to have cirrhosis. HCC and chronic renal disease were more prevalent in the DM group. Treatment uptake and SVR were comparable between groups. Regression analysis revealed that age and employment were associated with achieving SVR. Post-SVR HCC was higher in DM group.

**Conclusion:**

The high prevalence of DM in our HCV cohort supports screening. Further assessment is required to determine if targeted, early DAA treatment reduces DM onset, progression to cirrhosis and HCC risk. Further studies are needed to determine if optimization of glycemic control in this population can lead to improved liver outcomes.

## Summary

The interplay between HCV, DM, and DAA therapy is poorly understood. We compared HCV infection characteristics, treatment uptake, and treatment outcomes in patients with and without DM. Nearly 10% of patients with HCV had DM. These individuals were older, more likely have cirrhosis and possessed more comorbidities. DM patients were less likely to report unhealthy behaviours**.** Treatment uptake and SVR rates were comparable between both DM and non-DM groups.

## Introduction

Hepatitis C virus (HCV) infects over 100 million people and is a leading cause of liver-related morbidity and mortality [1, 2]. Diabetes mellitus (DM) affects an estimated 425 million people and can result in serious health complications [3]. Insulin resistance and DM adversely impact the course of liver disease, accelerating the rate of hepatic fibrosis and increasing the risk of hepatocellular carcinoma (HCC) [4, 5]. DM may also impact the likelihood of achieving sustained virological response (SVR) with HCV antiviral therapy [6–8]. With regard to diminished interferon treatment efficacy, insulin resistance impairs TNF signalling, thus blocking STAT-1 translocation and interferon-stimulated gene production which is critical to HCV antiviral activity [9].

DM is an extrahepatic complication of chronic HCV infection [10–16]. Evidence suggests that insulin resistance is the primary mechanism by which HCV induces DM [17–21]. According to experimental and clinical studies, HCV may interact with glucose metabolism to promote insulin resistance through various pathogenic processes: direct inhibition of insulin signalling [22], decreased incretin hormones [23], overproduction of proinflammatory cytokines [24, 25], oxidative stress [26], and pancreatic ß-cell dysfunction [27, 28].

Improved glycemic control may be attained in individuals achieving SVR with direct acting antiviral (DAA) therapy [29–31]. Publications have demonstrated early declines in fasting blood glucose and glycated hemoglobin during [32] and after [33] HCV treatment. Furthermore, the results of a prospective case-control study showed an improvement in pancreatic β-cell function following the clearance of HCV by DAAs [34]. Although the exact effects of HCV clearance on DM are unresolved, they are thought to involve alterations in glucose metabolism and insulin signalling [35, 36]. Suggested mechanisms include interrupting inflammatory pathways to restore glucose homeostasis [37] and increasing the hepatic expression of insulin receptor substrates [38].

In an effort to further evaluate the interplay between HCV, DM, and DAA therapy, we evaluated predictors of DM in a cohort of people living with chronic HCV. Furthermore, we compared HCV infection characteristics, treatment uptake, and treatment outcomes between patients with and without DM in our cohort receiving care at a regional HCV referral center.

## Methods

### Study design and population

We conducted a retrospective cohort study of patients assessed by The Ottawa Hospital Viral Hepatitis Program (TOH) between January 2014 and March 2020 (Ottawa Health Science Network Research Ethics Board #2004-196). TOH is a publicly funded, multidisciplinary program that provides comprehensive HCV care to individuals in Eastern Ontario, Western Quebec, and Nunavut, Canada. Consenting patients 18 years of age and older with chronic HCV infection (defined as HCV RNA positive at least 6 months after acute infection) were included in this analysis. Data from patient medical records was abstracted using standardized data collection forms and entered in the TOH Viral Hepatitis Program Database.

### Independent variables

DM status served as the primary independent variable. DM was defined as a diagnosis of either type 1 or type 2 DM as reported within patient health records. Covariates included (a) baseline characteristics (age at first clinical assessment, year of first visit to the clinic, sex, race, immigration status, HCV genotype and fibrosis stage); (b) baseline laboratory values (HCV RNA, hemoglobin, platelets, aspartate aminotransferase (AST), alanine aminotransferase (ALT), bilirubin, creatinine, liver stiffness in kilopascals, Control Attenuation Parameter (CAP score)); (c) social determinants of health (employment status, alcohol use history, incarceration history, injection drug use (IDU) history, and recreational drug use history); and (d) comorbidities (HIV co-infection, psychiatric illness, chronic renal disease, and HCC). Liver stiffness was assessed by transient elastography (Fibroscan) where a measure over 12.5 kPa was deemed evidence of cirrhosis. Fibroscan results were converted to the METAVIR scoring system where “F0” signified no fibrosis and “F4” signified cirrhosis [39]. CAP score was measured in dB/m by transient elastography where a result of 300 dB/m or more was considered evidence of steatosis [40].

### Dependent variables

Dependent variables included (1) Predictors of DM, (2) HCV treatment uptake and (3) HCV treatment outcomes as a function of DM status. HCV treatment initiation was defined as the provision of a DAA prescription. If more than one course of therapy was received, then the most recent round of therapy was considered. HCV treatment outcomes were evaluated in accordance to two main variables. The first was SVR, defined as HCV RNA negativity 12 weeks or more post-treatment. Crude SVR was calculated among patients who received at least one dose of DAA therapy. A treatment completion analysis was used to determine SVR among patients who completed a DAA therapy course and had post-treatment HCV RNA testing results available. The second was the incidence of newly diagnosed HCC cases at 12 and 24 months post-treatment. The enrollment of new participants was censored in March 2019. However, HCV treatment uptake and treatment outcomes data were collected until March 2020.

### Statistical analysis

All statistical analyses were performed using SAS Enterprise Guide (version 9.4m SAS Institute, Cary, NC). The distribution of baseline sociodemographic, laboratory, and clinical measures by patient DM status were described using the respective means (standard deviation [SD]) and proportions. Statistical comparisons between the two groups were made using χ^2^ tests for categorical variables and t-tests for continuous variables. Characteristics of patients who initiated and did not initiate DAA therapy were assessed by stratifying participants in accordance with DM status. Predictors of DM and SVR were assessed by logistic regression analysis adjusted for covariates and potential confounders. All significance tests were two-sided and *p*-values <0.05 were deemed statistically significant.

## Results

### HCV infection characteristics

One thousand five hundred and eighty-eight HCV-infected patients were included in this analysis. One hundred and fifty-three (9.6%) were diagnosed with DM (Table 1). Numerically, DM patients were more likely to be male (70.6%), between the ages of 60-69 years (38.6%) and Black (9.8%) at baseline assessment. Patients with DM were older, more likely to have advanced fibrosis and more likely to be infected with HCV genotypes 1b, 2, and 4. Only age predicted a diagnosis of DM by multivariate analysis (Table 2).

**Table 1:** Baseline characteristics and HCV treatment outcomes compared between DM and non-DM patients

**Baseline characteristics**	**Overall** *n*=1,588	**DM** *n*=153	**No DM** *n*=1,435	***p* value**
Age at first assessment, mean (SD)	51.0 (13.6)	58.1 (9.6)	50.3 (13.5)	<0.001
Age category, n (%)				
≤29	134 (8.4)	---	134 (9.3)	<0.001
30-39	185 (11.7)	4 (2.6)	181 (12.6)	
40-49	291 (18.3)	24 (15.7)	267 (18.6)	
50-59	532 (33.5)	51 (33.3)	481 (33.5)	
60-69	365 (23.0)	59 (38.6)	306 (21.3)	
≥70	81 (5.1)	15 (9.8)	66 (4.6)	
Male sex, n (%)	1,010 (63.6)	108 (70.6)	902 (62.9)	0.059
Race, n (%)^a^				
Caucasian	1,064 (83.3)	106 (79.1)	958 (83.8)	0.205
Black	79 (6.2)	13 (9.8)	66 (5.8)	
Asian	46 (3.6)	3 (2.2)	43 (3.8)	
Indigenous	69 (5.4)	9 (6.7)	60 (5.2)	
Hispanic	4 (0.3)	---	4 (0.4)	
Other	15 (1.2)	3 (2.2)	12 (1.0)	
HCV RNA (IU/mL), mean (SD)	3.9x10^6^ (13.3x10^6^)	3.3 x10^6^ (6.5x10^6^)	3.9 x10^6^ (13.9x10^6^)	0.384
Genotype, n (%)^a^				
G1a	693 (51.4)	63 (46.1)	630 (52.0)	0.010
G1b	113 (8.4)	15 (10.9)	98 (8.1)	
G1 (other subtypes)	46 (3.4)	2 (1.5)	44 (3.6)	
G2	99 (7.3)	15 (10.9)	84 (6.9)	
G3	293 (21.7)	26 (19.0)	267 (22.0)	
G4	52 (3.8)	15 (10.9)	37 (3.0)	
G5	3 (0.2)	1 (0.7)	2 (0.2)	
G6	8 (0.6)	---	8 (0.7)	
G mixed	42 (3.1)	---	42 (3.5)	
Liver stiffness (kPa), mean (SD)	11.2 (11.8)	18.3 (15.6)	11.2 (11.1)	<0.001
CAP score (dB/mL), mean (SD)	246 (61)	262 (71)	244 (60)	0.001
Fibrosis Stage, n (%)^a^				
F0-F1	766 (52.1)	35 (24.6)	731 (55.0)	<0.001
F2	197 (13.4)	16 (11.3)	181 (13.6)	
F3	172 (11.7)	25 (17.6)	147 (11.1)	
F4^b^	335 (22.8)	66 (46.5)	269 (20.3)	
Year of assessment, n (%)				
2014	239 (15.0)	24 (15.7)	215 (15.0)	0.552
2015	292 (18.4)	23 (15.0)	269 (18.7)	
2016	289 (18.2)	29 (18.9)	260 (18.1)	
2017	244 (15.4)	37 (24.2)	207 (14.4)	
2018	243 (15.3)	21 (13.7)	222 (15.5)	
2019	230 (14.5)	14 (9.2)	216 (15.1)	
2020	51 (3.2)	5 (3.3)	46 (3.2)	
**Baseline Laboratory Measures**				
ALT (IU/L), mean (SD)	92 (104.2)	92 (101)	91 (105)	0.922
AST (IU/L), mean (SD)	66 (66.9)	67 (72)	66 (66)	0.859
Bilirubin (mmol/L), mean (SD)	11 (10.0)	13 (16.5)	11 (8.6)	0.157
Hemoglobin (g/L), mean (SD)	140 (20)	137 (19)	140 (20)	0.031
Platelets (10^9^/L), mean (SD)	208 (81)	197 (88)	209 (80)	0.073
Creatinine (umol/L), mean (SD)	77 (71)	95 (98)	75 (66)	0.016
**Comorbidities**				
HIV co-infection, n (%)	61 (3.8)	6 (3.9)	55 (3.8)	0.957
Psychiatric condition, n (%)	751 (47.3)	77 (50.3)	674 (47.0)	0.429
Hepatocellular carcinoma, n (%)^a^	37 (2.3)	9 (5.9)	28 (1.8)	0.001
Chronic renal disease, n (%)^a^	34 (2.3)	20 (13.1)	14 (1.0)	<0.001
**Socioeconomic determinants of health**				
Immigrant, n (%)	269 (16.9)	44 (28.8)	225 (16.7)	<0.001
Currently employed, n (%)	305 (28.6)	35 (26.5)	270 (28.9)	0.564
Alcohol use history, n (%)	733 (55.7)	72 (47.1)	661 (56.9)	0.013
Incarceration history, n (%)^a^	634 (49.0)	58 (38.2)	576 (50.4)	0.006
IDU history, n (%)^a^	813 (62.4)	83 (54.6)	730 (63.5)	0.047
Recreational drug use history, n (%)^a^	749 (58.2)	69 (45.4)	680 (59.9)	0.007
**HCV treatment outcomes**				
DAA therapy initiated, n (%)				
Yes	1,072 (67.5)	133 (86.9)	939 (65.4)	<0.001
No	516 (32.5)	20 (13.1)	496 (34.6)	
Crude SVR rate, n (%)	831/995 (83.6)	118/131 (90.1)	713/864 (86.8)	0.032
Post treatment testing SVR rate, n (%)	831/846 (98.2)	118/122 (96.7)	713/724 (98.5)	0.173
Newly diagnosed hepatocellular carcinoma post treatment, n (%)				
12 months post treatment	3 (0.3)	1 (0.8)	2 (0.2)	---
24 months post treatment	8 (0.8)	3 (2.3)	5 (0.6)	---

^a^Denominator represents n_total_ – n_missing data_

^b^Cirrhosis by FibroScan defined as >12.5 kPa

**Table 2:** Predictors of diabetes diagnosis at baseline

**Effect**	**Unadjusted OR, 95% CI**	**Adjusted OR, 95% CI**
Age	1.05 (1.04-1.07)	1.06 (1.04-1.08)
Race^a^		
Black	1.78 (0.95-3.33)	1.30 (0.56-2.99)
Indigenous	1.36 (0.65-2.81)	1.33 (0.55-3.25)
Other	0.92 (0.39-2.18)	0.69 (0.22-2.16)
Caucasian	Ref.	Ref.
Male sex	1.42 (0.98-2.04)	1.31 (0.85-2.01)
Genotype^a^		
G1	0.58 (0.32-1.03)	0.87 (0.39-1.97)
G2	0.99 (0.46-2.13)	1.44 (0.55 -3.77)
G3	0.54 (0.28-1.06)	1.13 (0.46-2.78)
Other	Ref.	Ref.
HIV	1.02 (0.43-2.42)	0.97 (0.37-2.58)

^a^Denominator represents n_total_ – n_missing data_

The prevalence of several social determinants of health differed between DM and non-DM groups (Table 1). In the DM group there was a lower prevalence of alcohol use, incarceration, IDU and recreational drug use histories. Certain differences were observed in the proportion of concurrent co-morbidities between groups (Table 1). DM patients had a higher prevalence of chronic renal disease and HCC at baseline assessment. Individuals with DM were more likely to be cirrhotic at the time of DAA initiation. Mean baseline CAP score was higher in DM patients suggesting a greater burden of non-alcoholic steatohepatitis. Of note, the prevalence of HIV co-infection and psychiatric illness were similar.

### Treatment initiation

Of 1,588 patients, 1,072 (67.5%) initiated DAA therapy during the period of evaluation (Table 1). The initiation of DAA treatment was higher in the DM group compared with non-DM group (86.9% versus 65.4%, *p*<0.001). Within patients with DM, patient characteristics were similar between those who started DAA treatment and those who did not (data not shown). In contrast, initiating DAA in non-DM group was associated with older mean baseline age, genotype 1 infection, cirrhosis, Caucasian race, history of alcohol use and history of psychiatric illness (data not shown).

### Treatment outcomes

Crude SVR was comparable between DM (90.1%) and non-DM groups (86.8%) (Table 1). Likewise, SVR in those completing post-treatment HCV RNA testing was high and similar by group (96.7% vs 98.5%). By univariate analysis, DM status, older age and being employed were associated with achieving SVR (Table 3). After adjustment, age and employment status remained significant predictors of SVR. Of note, 77% of our DM group were on diabetes medications at the time of treatment. In an exploratory analysis, pharmacological treatment for diabetes did not impact SVR. The presence of steatosis as measured by transient elastography CAP score did not influence SVR.

**Table 3:** Predictors of Sustained Virologic Response in HCV treatment recipients

**Effect**	**Unadjusted OR, 95% CI**	**Adjusted OR, 95% CI**
DM diagnosis	1.09 (1.05-3.47)	1.37 (0.54-3.46)
Age at first assessment	1.03 (1.02-1.05)	1.02 (1.01-1.05)
Male sex	0.82 (0.57-1.18)	1.54 (1.33-2.72)
Genotype		
G1	0.65 (0.32-1.35)	1.48 (0.58-3.79)
G2	0.64 (0.25-1.66)	1.43 (0.78-2.61)
G3	0.51 (0.24-1.12)	0.38 (0.09-1.70)
Other	Ref.	Ref.
Cirrhosis by FibroScan^a^	1.17 (0.79-1.72)	1.40 (0.71-2.76)
Steatosis by Fibroscan^b^	1.26 (0.71-2.23)	1.44 (0.74-2.80)
Race		
Caucasian	1.14 (0.78-1.67)	0.74 (0.34-1.62)
Non-Caucasian	Ref.	Ref.
HIV co-infection	1.15 (0.48-2.88)	1.49 (0.48-4.65)
Psychiatric illness	0.95 (0.68-1.33)	0.59 (0.33-1.04)
Hepatocellular carcinoma	0.86 (0.37-2.00)	1.05 (0.13-8.74)
Currently employed	2.24 (1.32-3.81)	2.10 (1.06-4.15)
History of alcohol use	0.77 (0.54-1.11)	1.17 (0.66-2.09)
Incarceration history	0.77 (0.54-1.11)	0.56 (0.31-1.03)
IDU history	0.70 (0.48-1.03)	0.55 (0.28-1.10)

Denominator represents n_total_ – n_missing data_

^a^Cirrhosis by transient elastography defined as >12.5 kPa

^b^Steatosis by transient elastography defined as Controlled Attenuation Parameter score >300 dB/m

Although our analysis was limited by low frequency of events, the prevalence of newly diagnosed HCC was numerically higher in the DM group compared to the non-DM group at both 12 (0.8% versus 0.2%) and 24 (2.3% versus 0.6%) months post-treatment.

## Discussion

Our analysis utilized data from a cohort of HCV patients of which nearly 10% had DM. This proportion is within previously reported estimates ranging from 5.9-43.2% among HCV cohorts [41–43]. It also aligns with the global and Canadian DM prevalence among the general population (9.3%) [3, 44]. As a regional comparison, the prevalence of diabetes in Ontario was estimated to be 8.7% in 2016 [45]. Older age predicted DM in our cohort of people living with chronic HCV but not HCV genotype or race (Table 2). The prevalence of impaired fasting glycemia and DM has been demonstrated to increase with aging [46, 47]. Several groups have speculated that this is largely due to the age-related decrease in beta cell proliferation and enhanced sensitivity to apoptosis [48].

Our demographic results were comparative to those of other studies that reported HCV-infected with DM to be older [49–51] and numerically more likely to be non-Caucasian [51, 52] relative to non-diabetes patients (Table 1). DM patients in our cohort were more likely to be infected with genotypes 1b, 2, and 4. These findings are only partially in agreement with other studies who report higher frequencies of genotypes 1 [53], 3a [53–56] and 4 [53, 56] among DM patients. In this study, DM were less likely to engage in unhealthy lifestyle behaviours relative to non-diabetes patients. Similar observations have been made in Canadian studies comparing the behaviours of immigrant and non-immigrant patients with HCV [57–59]. Of note, there was a higher proportion of immigrants in the DM group.

A greater prevalence of baseline HCC and chronic renal disease was noted in those with DM. The higher mean age of our DM group may, at least in part, explain this finding. Another explanation is the pathophysiological association between DM, HCV, and chronic renal disease [60–64]. We found the prevalence of HIV co-infection to be similar in DM and non-DM patients. This result was unexpected as it contradicts previous studies that report DM to be more common in HIV-HCV co-infection [43, 60, 65]. A possible explanation for this finding is that the HIV patients included in our cohort were younger. We also found the prevalence of psychiatric illness to be comparable between the groups. This result is in contrast with other evidence that suggests an association between DM and mental health conditions [66–69]. The relationship between HCV, DM, and comorbidities may be partially attributed to psychosocial factors such as socioeconomic status. Given that we did not capture data in this realm, we were unable to explore how patient socioeconomic status influenced our findings.

In this cohorts there was a higher prevalence of cirrhosis in the diabetes group. This observation is consistent with previous literature [70, 71] and supports researchers that have found insulin resistance to accelerate fibrogenesis in HCV-infected subjects [72, 73]. DM is also associated with steatosis which also contributes to liver fibrosis [74]. It is noteworthy that the CAP score, a measure of liver steatosis, was greater among diabetes patients.

Collectively, the heightened risk of HCC, chronic renal disease and cirrhosis among patients with diabetes suggests that DM is linked to worse baseline health among patients with HCV. Santos speculated that this may be understood by considering the age at which HCV-infected patients with diabetes engage in care. In a prospective cohort analysis, DM was revealed to independently predict delayed access to HCV services. This represents a paradox, whereby people who are already at higher risk of advanced fibrosis are those that present late to care - a scenario that increases their risk of accelerated disease progression. The paradox is further complicated by the fact that DM is more prevalent within low socioeconomic groups [75]. These findings emphasize the need to pursue more timely testing and treatment for HCV among diabetes patients. As DM is more prevalent in socially deprived groups, specific screening strategies are recommended to address HCV within these subpopulations.

We found a greater proportion of DM patients initiated DAA therapy. This finding is likely confounded by cirrhotic state as patients with cirrhosis are more likely to be prescribed treatment for HCV; especially in the early era of DAA therapy when reimbursement was often dictated by fibrosis stage.

Historically, insulin resistance and DM have served as negative predictors of SVR in patients receiving interferon-based HCV treatment [6–8]. Fortunately, studies consistently demonstrate that new DAA therapies yield high SVR in nearly all patients with chronic HCV [76–78]. Our data showed that SVR rates were high in both the DM and non-DM patients. We found that being employed was associated with achieving SVR. This result is expected, as it aligns with previous studies that report employment to predict healthy lifestyle behaviours [79] and adherence to treatment [80, 81]. We also found that increased age at first assessment was associated with achieving SVR. This may be explained by the fact that substance abuse and mental health issues among treatment-seeking, older adults are generally less prevalent and less severe [82, 83]. Additionally, older adults tend to report more social support, more adaptive coping, and fewer barriers to treatment, relative to younger patients [82, 84]. Of note, genotype, cirrhosis and steatosis were not identified as predictors of SVR in DAA recipients.

We found that DM patients had a greater prevalence of newly diagnosed HCC after completing DAA therapy. Though limited by low frequency of events, these results suggest that HCC is more likely to develop in DM patients, even after HCV infection is cleared. Several studies have sought to investigate the incidence of HCC in cured HCV patients [85–88]. DM has rarely been found to predict HCC in individuals who achieve DAA-induced SVR [89]. Rather, reports suggest that older age [90] and baseline cirrhosis [90, 91] are predictors of HCC development post-treatment. This could explain why we observed an increased prevalence of HCC in diabetes patients with viral clearance as individuals in this group were older with more advanced fibrosis prior to initiating therapy. The increased prevalence of HCC post-SVR emphasizes the importance of early HCV treatment to prevent the development of initial cirrhosis as well as the need the maintain long-term, post-treatment HCC surveillance in patients with baseline cirrhosis [92, 93].

Several limitations are recognized in this retrospective study. Our analysis could be biased by missing data. However, there was minimal missingness for core variables including sex, age, comorbidities and fibrosis assessment. We were not able to account for patients with pre-diabetes (IFG/IGT) and undiagnosed diabetes. We were also unable to differentiate between type 1 and type 2 diabetes and how these may impact liver outcomes differently as type 1 is due to insulin deficiency as opposed to insulin resistance. However, the vast majority had type 2 diabetes. Sociodemographic data was self-reported. This could have introduced some degree of bias, particularly with respect to histories of incarceration, alcohol, and drug use. We were unable to consider certain variables that may affect the association between DM, HCV and DAA therapy including central obesity, glycemia control, insulin resistance-related inflammatory effects and specific diabetic medications [94, 95].

## Conclusions

DM is at least as common in those living with HCV as in the general population. This high prevalence as well as the burden of cirrhosis and HCC in this population support current calls for diabetes screening in those living with HCV. HCV screening should be considered in diabetic patients as well. We speculate that earlier HCV detection, treatment and cure could prevent progression to cirrhosis and reduce HCC risk. Further assessment is required to determine if targeted, early DAA treatment with cure may also reduce DM incidence. Additionally, studies are needed to determine if early diagnosis of DM and optimization of glycemic control may also help to modify HCV complication and improve liver outcomes in this population.

## Data Availability

The datasets generated and/or analysed during the current study are not publicly available due to privacy considerations but are available from the corresponding author on reasonable request.

## Abbreviations

DAA: Direct Acting Antiviral
DM: Diabetes mellitus
HCC: Hepatocellular carcinoma
HCV: Hepatitis C virus
IDU: Intravenous drug use
SVR: Sustained virological response
TOH: The Ottawa Hospital
